# Oral, genital and anal human papillomavirus infections among female sex workers in Ibadan, Nigeria

**DOI:** 10.1371/journal.pone.0265269

**Published:** 2022-03-30

**Authors:** Imran O. Morhason-Bello, Kathy Baisley, Miquel A. Pavon, Isaac F. Adewole, Rasheed A. Bakare, Silvia de Sanjosé, Suzanna C. Francis, Deborah Watson-Jones

**Affiliations:** 1 Obstetrics and Gynaecology Department, Faculty of Clinical Sciences, College of Medicine, University of Ibadan, Ibadan, Nigeria; 2 Institute of Advance Medical Research and Training, College of Medicine, University of Ibadan, Ibadan, Nigeria; 3 Clinical Research Department, Faculty of Infectious and Tropical Diseases, London School of Hygiene and Tropical Medicine, London, United Kingdom; 4 Department of Infectious Disease Epidemiology, Faculty of Epidemiology and Population Health, London School of Hygiene and Tropical Medicine, London United Kingdom; 5 Infection and Cancer Laboratory, Cancer Epidemiology Research Program, ICO, Bellvitge Biomedical Research Institute (IDIBELL), Centro de Investigación Biomédica en Red de Epidemiología y Salud Pública (CIBERESP), Barcelona, Spain; 6 Centro de Investigación Biomédica en Red de Epidemiología y Salud Pública (CIBERESP), Madrid, Spain; 7 Department of Microbiology, Faculty of Basic Medical Sciences, College of Medicine, University of Ibadan, Ibadan, Nigeria; 8 Division of Cancer Epidemiology and Genetics (DCEG), Consultant, National Cancer Institute (NCI), National Institutes of Health (NIH), USA and Associate Researcher, ISGlobal, Barcelona, Spain; 9 International Statistics and Epidemiology Group, London School of Hygiene and Tropical Medicine, London, United Kingdom; 10 Mwanza Intervention Trials Unit, National Institute for Medical Research, Mwanza, Tanzania; Istituto Nazionale Tumori IRCCS Fondazione Pascale, ITALY

## Abstract

**Background:**

There are limited data on the epidemiology of HPV in different anatomical sites of female sex workers (FSW). We investigated the prevalence and concordance of cervical, vulval, oral and anal HPV among FSW in Ibadan, Nigeria.

**Methods:**

FSWs aged 18–45 years were enrolled in a cross-sectional survey. After interview and clinical examination, samples were collected from mouth, cervix, vulva and anus. HPV genotyping was done with Anyplex II 28HPV assay. Multivariable analyses were performed to explore associated risk factors and concordance of HPV infections across sites.

**Results:**

In total, 315 FSWs participated in the study with a mean age of 30–6.5 years. The prevalence of any HPV infection was 88% in the vulva, 84% in the cervix, 75% in the anus and 24% in the oral cavity. HPV 35 was the most prevalent and concordant high-risk type in the four sites. The risk factors for HPV infection by anatomic site varied.

**Conclusion:**

This large study showed a high prevalence and concordance of HPV infections of cervical, vulval, oral and anal HPV among FSWs in Nigeria. The potential to acquire and transmit HPV is high in this population, and we highlighted the urgency to protect young women through HPV vaccination.

## Introduction

Human papillomavirus (HPV) infection occurs in nearly 80% of sexually active people at least once in a lifetime [1, 2]. Most HPV infection will clear but it may persist in 10% of people leading to warts and HPV-associated cancers [2]. The highest burden of morbidity and mortality from HPV infection is in low middle-income countries, especially, in sub-Saharan Africa (SSA) [3]. Female sex workers (FSWs) engage in high sexual risk behaviours such as frequent unprotected sexual acts, multiple sexual partnerships, and substance abuse. Challenges with negotiating safe sex with clients, and financial inducement to engage in risky behaviours [4–6] make FSWs vulnerable to acquiring sexually transmitted infections (STI), including HPV compared to women in the general population.

FSWs have more than two-times the risk of acquiring genital HPV infections and increased prevalence of abnormal pap smears than women in the general population [7]. In 2020, a meta-analysis of 62 studies involving 21,402 FSWs from 33 countries showed a wide range of prevalence of genital HPV infections (5.5–84.7%) [8]. In Senegal, 79.8% of FSW had vaginal HPV infections and 70.1% of those with HPV infection had multiple genotypes [9]. In Togo, 45.2% and 34.8% of FSWs had cervical and anal HPV infections respectively [10]. In the same study, high-risk (HR) cervical and anal HPV infections were more frequent among HIV positive FSWs relative to HIV negative FSWs, and 43.2% of those with HR-HPV infections had the same genotypes in the cervix and anal cavity [10].

In 2019, a systematic review showed that sexually active women including FSWs engage in heterosexual oral and anal sex in SSA [11]. However, there is no published study in SSA that assess prevalence of oral and vulvar HPV infections in FSWs. This study investigates the prevalence and concordance of HPV in the oral cavity, anus, vulva and cervix. This information would help to further understand the burden HPV and to design future preventive strategies.

## Materials and methods

### Study design, population, and study site

This cross-sectional survey was conducted among FSWs aged 18–45 years in brothels as part of the Sexual behaviour and HPV Infection in Nigerians in Ibadan (SHINI) study [12]. Brothels were selected from the six urban local government areas (LGAs) in Ibadan: Ibadan North, Northwest, Northeast, Southwest, and Southeast and Akinyele.

### Study procedures

#### Sampling and enrolment of study participants

Before enrolment, trained field workers mapped and updated the existing list of brothels in the six urban LGAs in Ibadan. Eligible participants were listed and assigned a unique number by female research assistants by questioning the manager/chairlady (leader of FSWs). For brothels that had 10 FSWs or fewer, we invited all eligible FSWs while 90% of the FSWs were selected by a simple random sampling in brothels that had 11 or more eligible participants. Female research assistants visited each brothel to explain the study objectives to potential participants collectively and then individually in their rooms, and a copy of an information leaflet was also given to each of them. After, a signed consent for was obtained which also covered storage of samples for future use.

#### Interview, clinical examination, sample collection and follow-up

Interview and sample collection were done inside the bedroom of each participant. A face-to-face interview with paper questionnaires was conducted in English or pidgin English for participants that could not understand English by female research assistants. The interview covered socio-demographics, sexual behaviours, condom use and hygiene practices, intravaginal practices, alcohol consumption, smoking, stimulant use, previous STI and awareness about HPV vaccine. After the interview, a female nurse collected blood and performed rapid diagnostic HIV test (RDT using Nigerian HIV test protocol [13]). Participants who tested HIV positive were referred to government designated HIV care centres for confirmation and management. We performed anonymous RDT to those unwilling to know their HIV status.

Biological samples were collected by a trained nurse from the cervix, vulva and oral and anal cavities in separate sample bottles. Briefly, an oral sample was collected using a 30 second oral rinse and gargle method with 10mls of Scope mouth wash (Procter & Gamble^©^). The nurse demonstrated the rinse and gargle procedure to individual participant. The participant sample was then collected into a 10 ml labelled sample bottle and placed immediately into a cold box (2–4°C). For the vulvar sample, the tip of a Dacron swab was used to rub the introitus on either side of the vaginal orifice without touching the urethral orifice. The cervical sample was then collected by inserting a sterile Cusco speculum into the vagina to expose the cervix. The tip of a new Dacron swab was inserted into the cervical os and gently rotated 360 degrees to avoid trauma to the cervix and potential bleeding before removing it. An anal sample was collected with the participant in a left lateral position. A Dacron swab was inserted into the anal canal (about 5–6 cm beyond the anal verge) and rotated 360 degrees with gentle pressure around the anal verge before removing it. Swab were placed in separate 2 ml cryotubes that were labelled and barcoded prior to being placed into a cold box (2–4°C).

All samples were stored in a -80°C freezer at the Institute of Advance Medical Research and Training, University of Ibadan. Nigeria. Each participant was provided with incentives for appreciation.

### HPV genotyping

DNA extraction from the mouthwash was performed using the Maxwell^®^ 16 LEV Blood DNA kit (Promega Corp., Madison, WI, USA), while cervical, vulvar and anal dry swabs were performed using the Maxwell 16 Buccal swab LEV DNA Purification kit, following the procedures previously described [12].

HPV genotyping of samples were performed at the Catalan Institute of Oncology, Spain using the Anyplex^™^ II HPV28 (Seegene, Seoul, South Korea) assay [14]. The Anyplex^™^ II HPV28 detection test was performed according to the manufacturer’s instructions using 10 μl of sample DNA [14]. Human Beta (β)-globin was used as the internal control (IC) in each sample to identify true HPV negative samples and distinguish it from those invalid HPV negative samples in which there is not enough biological material to carry out the HPV analysis. HPV negative samples with a positive result for the IC and HPV positive sample were classified as valid, while HPV negative samples no amplification of IC were classified as invalid samples.

### Data management

The interview data were double entered into REDCap software (*Vanderbilt University*, *Nashville Tennessee*, *USA*). The CSV data format were imported into STATA 16.0 software for analysis. Descriptive statistics were performed and presented. The primary outcome was prevalence of any HPV infection. The prevalence of HR-HPV and LR-HPV infection and by 2009 IARC epidemiological oncogenic classification for each of the anatomical sites were calculated. The trends of association between each classification of HPV infection and the age group of participants were calculated with ANOVA. The association between any HPV infection and explanatory variables were tested using hierarchical logistic model as shown in the conceptual framework (S1 Fig). Any HPV infection was treated as a binary outcome for each of four anatomical sites. Age group and study sites were included in the adjusted estimates *a priori*. Explanatory variables were grouped into Level 1 (sociodemographic factors), Level 2 (behavioural factors) and Level 3 (biological factors) (S1 Fig).

Each variable in Level 1 (sociodemographic factors) was added one by one to the model that included age group and study site (S1 Fig). P-values were obtained by likelihood ratio tests. Any variable that met a p value ≤ 0.10 was included in the adjusted model. This procedure was repeated for the analyses of behavioural (levels 2) and biological (level 3) variables, except that the multivariable models for these two levels were each adjusted for not only other respective level 2 and 3 factors with adjusted associations with p<0.10, but also for sociodemographic factors that were found to be independently associated with any HPV infections in level 1. The concordance of HPV between oral, cervical, vulvar and anal samples in individual participant was defined as the presence of the same type of virus across the four sites. The proportion of concordance for specific HPV type was calculated as the number of each HPV type in all the four sites, any of three and any of two anatomical sites.

### Ethics approvals

Ethical approvals were obtained from the ethical committees of the London School of Hygiene and Tropical Medicine, London (LSHTM 9736–3); the University of Ibadan/the University College Hospital, Ibadan (UI/EC/16/005); and the Oyo State Government (AD13/479/712) in Nigeria. Participants gave a written consent for participation and for their data to be published in a scientific journal.

## Results

**Fig 1:**
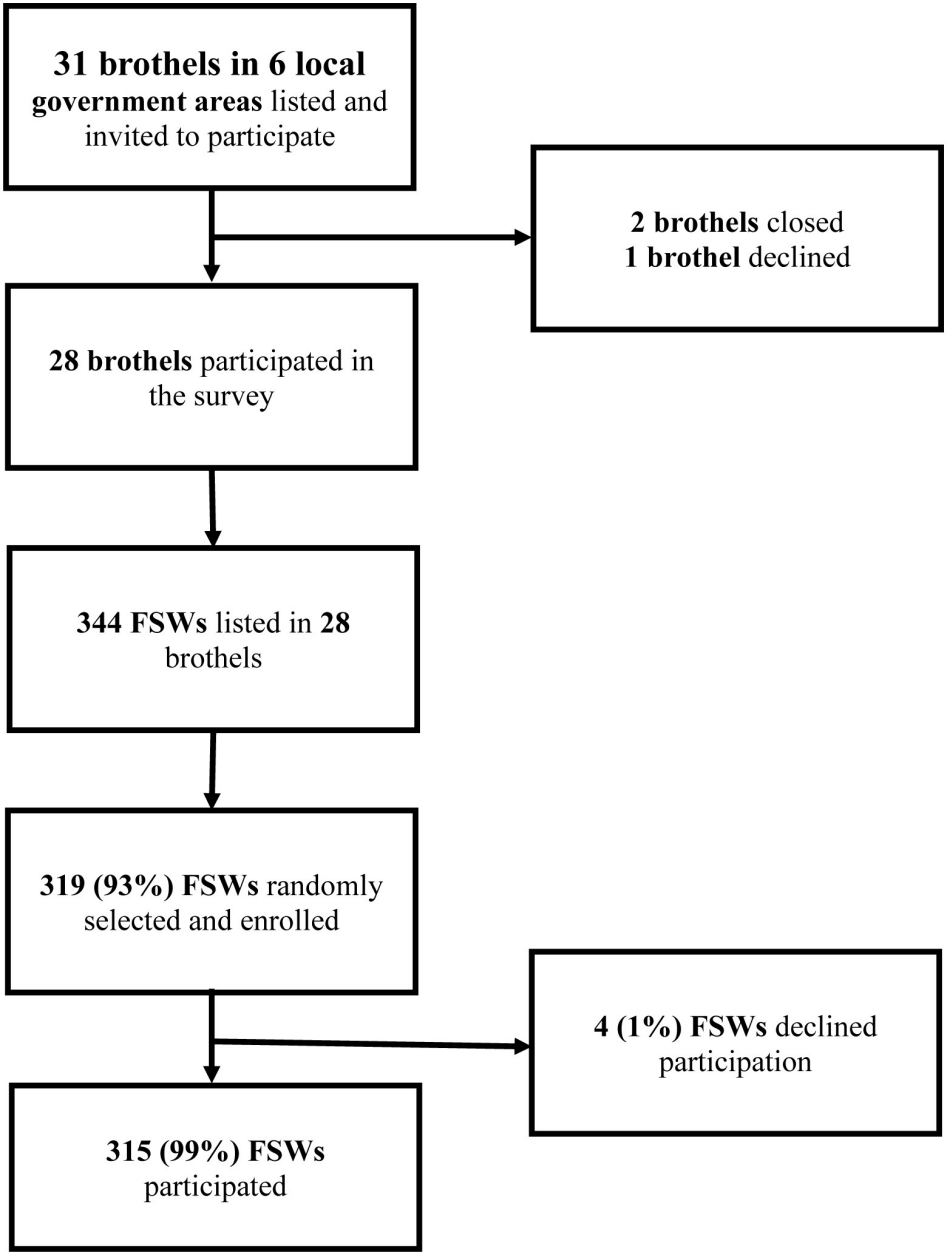
Brothel-based female sex workers enrolment flow for SHINI study in Ibadan, Nigeria.

Out of 31 brothels in the 6 LGAs, two had closed and the management of one brothel declined participation. Three hundred and forty-four FSWs were listed from the remaining 28 brothels; 319 FSWs were randomly selected proportionate to brothel size. Of these, 315 FSWs consented and 4(1%) declined to partook in the study (Fig 1).

### Participant characteristics

The mean age of FSWs was 30.4 years (SD = 6.5); 55% were 25 to 34 years old. The median age of vaginal sexual debut of participants and their partners was 18.0 years (inter quartile range = 4.0) and 23.0 years (Inter quartile range = 7.0), respectively. About a quarter had passed their sexual debut by 15 years. Most (94%) FSWs reported using condoms during their last vaginal sex act and 160 (51%) reported more than 50 vaginal sex partners in the past 3 months.

Sixty-one (19%) FSWs reported ever giving a male partner oral sex while 135 FSWs (43%) had ever received oral sex from a male partner. Only eight (3%) had received anal sex. A third (32%) were initiated into sex work by the age of 24 years. Fourteen (4%) FSWs had heard of HPV vaccine. Forty-two (13%) participants were HIV positive (Table 1).

**Table 1:** Socio-demographic characteristics of brothel-based female sex workers in Ibadan, Nigeria (n = 315).

Variable	Frequency	Percentage
***SOCIO-DEMOGRAPHIC FACTORS (Level 1)***		
**Age, years**		
Mean (SD)	30(SD = 6.5)	
**Age group, years**		
18–24	55	17%
25–34	172	55%
36–45	88	28%
**Ethnicity**		
Yoruba	30	10%
Hausa/Fulani	6	2%
Igbo	83	26%
Other ethnic minorities^1^	196	62%
**Religion** ^2^		
Christianity	286	91%
Islam	27	9%
**Highest education level**		
No formal education	21	7%
Primary	65	21%
Secondary	201	63%
Tertiary	28	9%
**Quranic education**		
Yes	21	7%
No	294	93%
**Occupation**		
No current paid job (e.g. student, apprentice, housewife)	226	72%
Unskilled worker (e.g. office assistant, food vendor)	4	1%
Semi-skilled worker (e.g. driver, caterer, tailor)	82	26%
Skilled worker (e.g. teacher, nurse)	3	1%
**Income per month** ^3^		
≤20,000N (≤56 USD)	37	12%
20,001–40,000N (>56–112USD)	176	56%
> 40,000N (>112USD)	102	32%
**Items personally owned by participant**		
Television	123	39%
Radio	63	20%
**Current marital status**		
Single^4^	143	45%
Married and living as married Divorced/Widowed^4^	172	55%
***BEHAVIOURAL FACTORS (Level 2)***		
**Age at first vaginal sex**^5^ **in years**		
≤ 15	78	26%
16–17	71	24%
≥ 18	153	51%
**Age difference between first vaginal sex partner and participant in years** ^6^		
0	21	8%
1–5	143	54%
≥ 6	100	38%
**Number of partners with vaginal sex in past 3 months** ^7^		
≤ 25	37	13%
26–50	72	27%
51–75	29	11%
76–100	67	25%
> 100	64	24%
**Condom use during last vaginal sex**		
No	18	6%
Yes	296	94%
**Ever gave oral sex to a male partner**		
No	254	81%
Yes	61	19%
**Ever received oral sex from a male partner**		
No	180	57%
Yes	135	43%
**Age initiated into sex work, year**		
≤19	24	8%
20–24	77	24%
25–29	100	32%
≥30	114	36%
**Years in commercial sex activity**		
<1	74	23%
1–<3	134	43%
3–<5	59	19%
≥5	48	15%
**Ever practised mutual masturbation** ^8^		
No	8	3%
Yes	307	97%
**Ever practised self-masturbation**		
No	100	32%
Yes	215	68%
**Female genital mutilation** ^9^		
No	185	59%
Yes	130	41%
**Ever drank alcohol**		
No	78	25%
Yes	237	75%
**Ever smoked cigarettes**		
No	214	68%
Yes	101	32%
**Ever taken any illicit drug** ^10^		
No	239	76%
Yes	76	24%
**Ever had an STI**		
No	204	65%
Yes	111	35%
**Ever heard of HPV**		
No	301	96%
Yes	14	4%
***BIOLOGICAL FACTORS (Level 3)***		
**Rapid diagnosis of HIV at the clinic**		
Positive	42	13%
Negative	273	87%
**Cervical HPV infection** ^8^		
No	50	16%
Yes	263	84%
**Vulvar HPV infection**		
No	38	12%
Yes	277	88%
**Anal HPV infection** ^9^		
No	79	25%
Yes	232	75%
**Oral HPV infection** ^10^		
No	214	76%
Yes	68	24%

^**1**^**-**Edo, TIV, Igbira;

^**2**^**-**N = 313 –two participants gave no response;

^**3**^**-** N—Naira-Nigeria currency; USD—United States Dollar;

^**4**^**-** Living alone;

^**5**^-N = 267–48 missing;

^**6**^-N = 302–13 missing;

^**7**^-N = 269–46 missing;

^**8**^- Mutual masturbation question was ‘have you or your partner ever touched each other’s genital area by hand? (Yes or No);

^**9**^-Female genital mutilation was based on the clinical examination of the female external genitalia for evidence of genital circumcision by the research nurse at the clinic (Yes or No);

^**10**^- Illicit drugs are banned substances or drugs taken by participants for non-medical reasons in Nigeria.

### Prevalence of cervical, vulvar, anal and oral HPV infection

Of the 1260 samples collected from the 4 anatomic sites, two (0.6%) cervical, four (1.3%) anal swabs, and 33 (10.5%) mouthwash samples were declared as invalid in the laboratory due to the lack of amplification of HPV and the internal control. These invalid samples were treated as missing variables. In total, 303 (96%) FSWs were positive for at least one HPV genotypes, 274 (87%) FSWs had one HR-HPV and 257 (82%) FSWs had one LR-HPV.

Overall, the prevalence of any HPV among the FSWs was 87.9% (95% CI, 83.8–91.3) in the vulva, 84.0% (95% CI, 79.5–87.9) in the cervix, 74.6% (95% CI, 69.4–79.3) in the anus and 24.1% (95% CI, 19.2–29.5) in the mouth (Table 2).

**Table 2:** Prevalence of Human papillomavirus infections among 315 brothel-based female sex workers in Ibadan, Nigeria.

Variable	Cervical Sample		Vulvar Sample		Anal Sample		Oral Sample	
	n/N^1^	Prevalence (%) [95% CI]	n/N	Prevalence (%) [95% CI]	n/N	Prevalence (%) [95% CI]	n/N	Prevalence (%) [95% CI]
**Any HPV genotypes**		**p = 0.041** ^2^		**p = 0.001** ^2^		p = 0.303		p = 0.112
18–24 years	51/55	92.7 (82.4–98.0)	54/55	98.2 (90.3–99.9)	48/55	87.3 (75.5–94.7)	16/46	34.8 (21.4–50.2)
25–34 years	145/171	85.0 (78.5–89.8)	154/172	89.5 (84.0–93.7)	125/171	73.1 (65.8–79.6)	37/154	24.0 (17.5–31.6)
35-45years	67/87	77.0 (66.8–85.4)	69/88	78.4 (68.4–86.5)	59/85	69.4 (58.5–79.0)	15/82	18.3 (10.6–28.4)
Overall	263/313	84.0 (79.5–87.9)	277/315	87.9 (83.8–91.3)	232/311	74.6 (69.4–79.3)	68/282	24.1 (19.2–29.5)
**HPV classification by IARC**^3^ *Class 1 –Carcinogenic*^4^		**p = 0.041**		**p<0.001** ^2^		**p = 0.016**		p = 0.125
18–24 years	44/55	80.0 (67.0–89.6)	51/55	92.7 (82.4–98.0)	42/55	76.4 (63.0–86.8)	8/46	17.4 (7.8–31.4)
25–34 years	111/171	64.9 (57.3–72.0)	118/172	68.6 (61.1–75.5)	94/171	55.0 (47.2–62.6)	25/154	16.2 (10.8–23.0)
35-45years	52/87	59.8 (48.7–70.1)	55/88	62.5 (51.5–72.6)	48/85	56.5 (45.3–67.2)	6/82	7.3 (2.7–15.2)
Overall	207/313	66.1 (60.6–71.4)	224/315	71.1 (65.8–76.1)	184/311	59.2 (53.5–64.7)	39/282	13.8 (10.0–18.4)
*Class 2A –Probable carcinogenic* ^5^		p = 0.407		p = 0.396		p = 0.122		p = 0.351^2^
18–24 years	11/55	20.0 (10.4–33.0)	13/55	23.6 (13.2–37.0)	11/55	20.0 (10.4–33.0)	2/46	4.3 (0.5–14.8)
25–34 years	35/171	20.5 (14.7–27.3)	34/172	19.8 (14.1–26.5)	17/171	9.9 (5.9–15.4)	2/154	1.3 (0.2–4.6)
35-45years	12/87	13.8 (7.3–22.9)	13/88	14.8 (8.1–23.9)	9/85	11.6 (5.1–21.6)	1/82	1.2 (0.03–6.6)
Overall	58/313	18.5 (14.4–23.3)	60/315	19.0 (14.9–23.8)	37/311	10.6 (5.0–19.2)	5/282	1.8 (0.6–4.1)
*Class 2B –Possible carcinogenic* ^6^		**p = 0.007**		**p<0.001** ^2^		p = 0.279		p = 0.144
18–24 years	45/55	81.8 (69.1–90.9)	50/55	90.9 (80.0–96.9)	34/55	61.8 (47.7–74.6)	9/46	19.6 (9.4–33.9)
25–34 years	109/171	63.7 (56.1–70.9)	120/172	69.8 (62.3–76.5)	85/171	49.7 (42.0–57.4)	16/154	10.4 (6.1–16.3)
35-45years	49/87	56.3 (45.3–66.9)	50/88	56.8 (45.8–67.3)	43/85	50.6 (39.5–61.6)	7/82	8.5 (3.5–16.8)
Overall	203/313	64.9 (59.3–70.1)	220/315	69.8 (64.4–74.9)	162/311	52.1 (46.4–57.8)	32/282	11.3 (7.9–15.6)
*Class 3 –Unclassified* ^7^		p = 0.532		p = 0.210^2^		p = 0.063^2^		p = 0.365^2^
18–24 years	7/55	12.7 (5.3–24.5)	11/55	20.0 (10.4–33.0)	11/55	20.0 (10.4–33.0)	0/46	0
25–34 years	28/171	16.4 (11.2–22.8)	30/172	17.4 (12.1–24.0)	19/171	11.1 (6.8–16.8)	6/154	3.9 (1.4–8.3)
35-45years	10/87	11.5 (5.7–20.1)	9/88	10.2 (4.8–18.5)	6/85	7.1 (2.6–14.7)	2/82	2.4 (0.3–8.5)
Overall	45/313	14.4 (10.7–18.8)	50/315	15.9 (12.0–20.4)	36/311	11.6 (8.2–15.7)	8/282	2.8 (1.2–5.5)
**Any HR-HPV genotypes** ^8^		**p = 0.008**		**p<0.001** ^2^		**p = 0.006**		p = 0.143
18–24 years	47/55	85.5 (73.3–93.5)	53/55	96.4 (87.5–99.6)	44/55	80.0 (67.0–89.6)	9/46	19.6 (9.4–33.9)
25–34 years	117/171	68.4 (60.9–75.3)	127/172	73.8 (66.6–80.2)	97/171	56.7 (48.9–64.3)	26/154	16.9 (11.3–23.8)
35-45years	53/87	60.9 (49.9–71.2)	57/88	64.8 (53.9–74.7)	48/85	56.5 (45.3–67.2)	7/82	8.5 (3.5–16.8)
Overall	217/313	69.3 (63.9–74.4)	237/315	75.2 (70.1–79.9)	189/311	60.8 (55.1–66.2)	42/282	14.9 (10.9–19.6)
**Any LR-HPV genotype** ^9^		p = 0.005		p<0.001		p = 0.241		p = 0.409
18–24 years	47/55	85.5 (73.3–93.5)	51/55	92.7 (82.4–98.0)	35/55	63.6 (49.6–76.2)	9/46	19.6 (9.4–33.9)
25–34 years	113/171	66.1 (58.5–73.1)	123/172	71.5 (64.1–78.1)	88/171	51.5 (43.7–59.2)	22/154	14.3 (9.2–20.8)
35-45years	52/87	59.8 (48.7–70.1)	51/88	58.0 (47.0–68.4)	43/85	50.6 (39.5–61.6)	9/82	11.0 (5.1–19.8)
Overall	212/313	67.7 (62.2–72.9)	225/315	71.4 (66.1–76.4)	166/311	53.4 (47.7–59.0)	40/282	14.2 (10.3–18.8)
**Multiple HPV genotypes** ^10^		**p = 0.001** ^2^		**p<0.001** ^2^		**p = 0.001**		p = 0.330
18–24 years	47/55	85.5 (73.3–93.5)	51/55	92.7 (82.4–98.0)	44/55	80.0 (67.0–89.6)	7/46	15.2 (6.3–28.9)
25–34 years	113/171	66.1 (58.5–73.1)	117/172	68.0 (60.5–74.9)	84/171	49.1 (41.4–56.9)	14/154	9.1 (5.1–14.8)
35-45years	48/87	55.2 (44.1–65.9)	53/88	60.2 (49.2–70.5)	44/85	55.1 (40.7–62.7)	6/82	7.3 (2.7–15.2)
Overall	208/313	66.5 (60.9–71.7)	221/315	70.2 (64.8–75.2)	172/311	55.3 (49.6–60.9)	27/282	9.6 (6.4–13.6)

^**1**^**-**n/N—number of samples with positive HPV infection as numerator and total samples with valid result as denominator;

^**2**^- Bartlett’s test for equal variances were significant (p< 0.05);

^**3**^**-IARC**–International Agency for Research on Cancer (*- HPV genotypes in IARC classification that are not included in the Anyplex II HPV28 platform);

^**4**^**- Class 1 IARC HPV** -16, 18, 31, 33, 35, 39, 45, 51, 52, 56, 58, 59;

^**5**^**- Class 2A IARC HPV**—68;

^**6**^**- Class 2B IARC HPV**—5*, 8*, 26, 30*, 34*, 40, 42, 43, 44, 53, 54, 55*, 61, 66, 67*, 69, 70, 71*, 72*, 73, 81*, 82, 83*, 84*, 85*, 97*, IS39* and CP6108*;

^**7**^**- Class 3 IARC HPV**—6, 11;

^**8**^**–14 HR-HPV Group**—Class 1 IARC HPV and Class 2A IARC HPV;

^**9**^–**14 LR-HPV**—Class 2b IARC and Class 3 IARC;

^**10**^**-** Multiple HPV infection- Detection of two or more genotypes of HPV by Anyplex II HPV28 from a sample; All invalid samples were excluded from the descriptive analysis.

**Fig 2:**
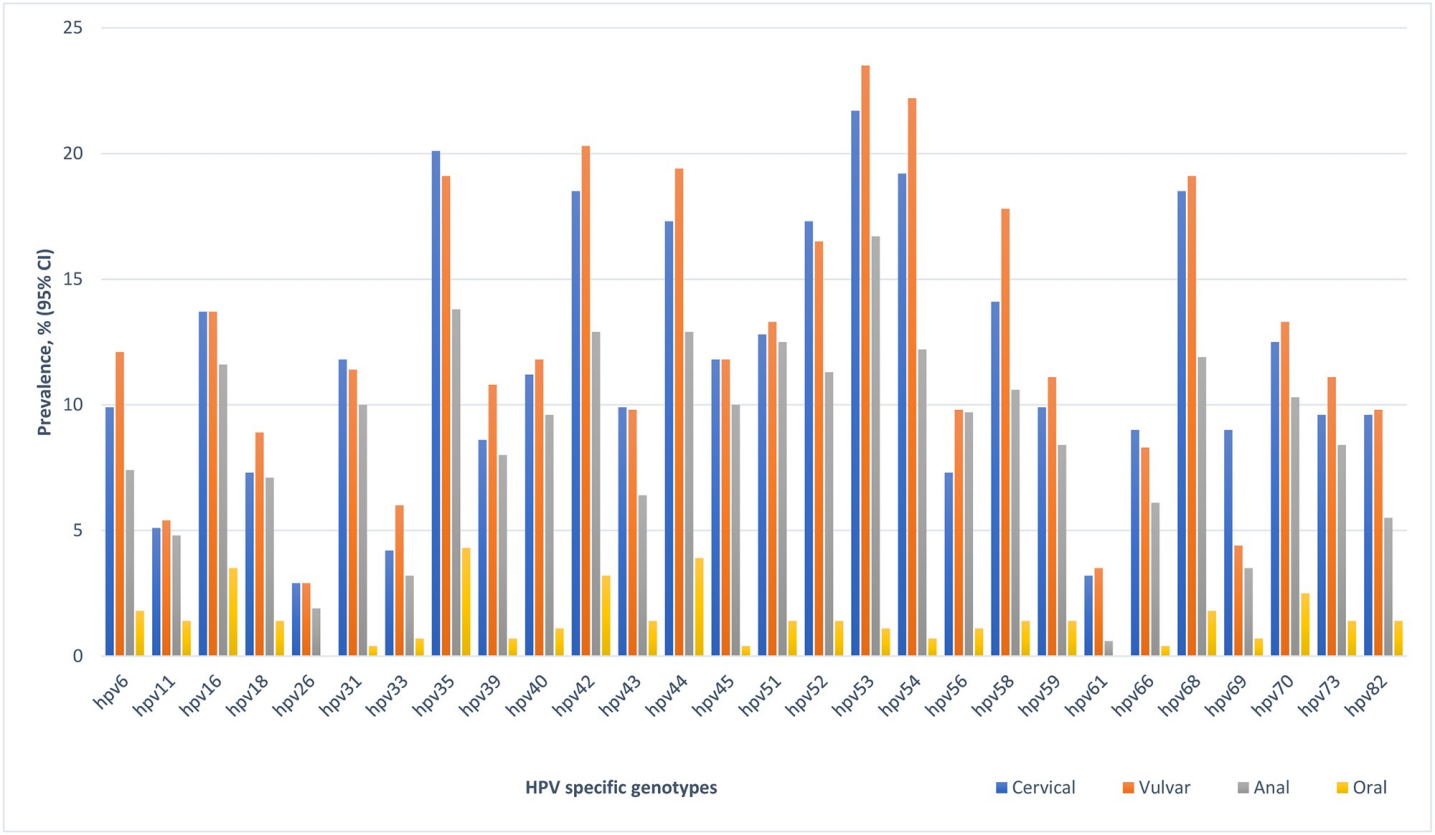
Prevalence of specific HPV genotypes according to the four anatomic sites among brothel based female sex workers in Ibadan Nigeria.

There was an inverse relationship between the age of participants and any HPV, HR-HPV, and multiple HPV at all anatomic sites. Specifically, there was a significant inverse association between any HPV and age of participants in the vulvar and cervical samples. There was no association with age and HPV detection in oral samples by any HPV classification (Table 2). HPV-35 was the most prevalent HR-HPV in the 4 anatomic sites (Fig 2; S2 Fig): vulva (20.1%), cervix (19.1%), anal cavity (18.2%) and oral cavity (4.3%). HPV-53 was the most common LR-HPV genotypes detected in the vulvar (23.5%), cervical (21.7%) and anal (16.1%) samples, and HPV-44 in oral (3.9%) sample.

### Risk factors associated with cervical, vulvar, anal and oral HPV infection

The results of crude and adjusted models for the risk factor analyses associated with each of the four anatomic sites were presented in Tables 3 and 4. Participants aged 25–34 years (adjusted odds ratio (aOR) = 0.44, 95% CI, 0.15–1.31) and 35–45 years (aOR = 0.26, 95% CI, 0.08–0.82) had lower odds of having cervical HPV compared to those aged 18–24 years. There was 0.48 times lower odds (aOR = 0.48, 95% CI, 0.26–0.89) of cervical HPV in women that had clinical evidence of female genital mutilation (FGM) than those without. However, being HIV positive was associated with 11.39 (95% CI, 1.03–125.83) times higher odds of having cervical HPV compared to those that were HIV negative, and those women who had any vulvar HPV had 16.60 (95% CI, 5.08–47.54) times higher odds of having cervical HPV than those without any vulvar HPV.

**Table 3:** Descriptive summaries and unadjusted factors associated with cervical, vulvar, anal and oral human papillomavirus infection among brothel-based female sex workers in Ibadan, Nigeria.

Variable	Cervical		Vulvar		Anal		Oral	
	n/N (row, %)	p-value^1^ Crude OR (95%CI)	n/N (row, %)	p-value^1^ Crude OR (95%CI)	n/N (row, %)	p-value^1^ Crude OR (95%CI)	n/N (row, %)	p-value^1^ Crude OR (95%CI)
***SOCIO-DEMOGRAPHIC FACTORS***								
**Age group, years**		**p = 0.035**		**p<0.001**		**p = 0.034**		p = 0.120
18–24	51/55 (93%)	**1**	54/55 (98%)	**1**	48/55 (87%)	**1**	16/46 (35%)	1
25–34	145/171 (85%)	**0.44 (0.15–1.31)**	154/172 (90%)	**0.16 (0.02–1.22)**	125/171 (73%)	**0.39 (0.17–0.94)**	37/154 (24%)	0.59 (0.29–1.21)
35–45	67/87 (77%)	**0.26 (0.08–0.82)**	69/88 (78%)	**0.07 (0.01–0.52)**	59/85 (69%)	**0.33 (0.13–0.83)**	15/82 (18%)	0.42 (0.18–0.96)
**Ethnicity**		p = 0.732		p = 0.306		p = 0.228		p = 0.644
Yoruba	25/29 (86%)	1	28/30 (93%)	1	25/30 (83%)	1	6/29 (21%)	1
Other^2^	238/284 (84%)	0.83 (0.28–2.49)	249/285 (87%)	0.49 (0.11–2.16)	207/281 (74%)	0.56 (0.21–1.52)	62/253 (25%)	1.24 (0.48–3.20)
**Religion**		p = 0.846		p = 0.761		p = 0.532		p = 0.908
Christianity	239/284 (84%)	1	251/286 (88%)	1	209/282 (74%)	1	61/254 (24%)	1
Islam and no religion^3^	24/29 (83%)	0.90 (0.33–2.49)	26/29 (90%)	1.21 (0.35–4.20)	23/29 (79%)	1.34 (0.52–3.42)	7/28 (25%)	1.05 (0.43–2.60)
**Highest education level**		p = 0.931		p = 0.526		p = 0.593		p = 0.242
No formal education	17/21 (81%)	1	17/21 (81%)	1	14/21 (67%)	1	8/19 (42%)	1
Primary	56/65 (86%)	1.46 (0.40–5.36)	59/65 (91%)	2.31 (0.58–9.51)	51/65 (78%)	1.82 (0.62–5.38)	13/60 (22%)	0.38 (0.13–1.14)
Secondary	167/199 (84%)	1.23 (0.39–3.89)	178/201 (89%)	1.82 (0.56–5.88)	146/199 (73%)	1.38 (0.53–3.60)	39/177 (22%)	0.39 (0.15–1.03)
Tertiary	23/28 (82%)	1.08 (0.25–4.64)	23/28 (82%)	1.08 (0.25–4.64)	21/26 (81%)	2.10 (0.55–7.96)	8/26 (31%)	0.61 (0.18–2.10)
**Quranic education**		p = 0.283		p = 0.703		p = 0.476		p = 0.649
No	248/293 (85%)	1	258/294 (88%)	1	215/290 (74%)	1	64/262 (24%)	1
Yes	15/20 (75%)	0.54 (0.19–1.57)	19/21(90%)	1.33 (0.30–5.93)	17/21 (81%)	1.48 (0.48–4.55)	4/20 (20%)	0.77 (0.25–2.40)
**Other occupation**		p = 0.407		p = 0.533		p = 0.485		p = 0.731
Student or Apprentice	194/228 (85%)	1	203/229 (89%)	1	171/226 (76%)	1	51/207 (25%)	1
Others^4^	69/85 (81%)	0.76 (0.39–1.45)	74/86 (86%)	0.79 (0.38–1.65)	61/85 (72%)	0.82 (0.47–1.43)	17/75 (23%)	0.90 (0.48–1.68)
**Income per month** ^5^		p = 0.855		p = 0.720		**p = 0.020**		p = 0.588
≤20,000N (≤56 USD)	83/98 (85%)	1	85/99 (86%)	1	63/98 (64%)	**1**	25/90 (28%)	1
20,001–40,000N (>56–112USD)	96/113 (85%)	1.02 (0.48–2.17)	102/114 (89%)	1.40 (0.61–3.19)	89/113 (79%)	**2.06 (1.12–3.80)**	21/98 (21%)	0.71 (0.36–1.38)
> 40,000N (>112USD)	84/102 (84%)	0.84 (0.40–1.78)	90/102 (88%)	1.24 (0.54–2.82)	80/100 (80%)	**2.22 (1.17–4.22)**	22/94 (23%)	0.79 (0.41–1.54)
**Current marital status**		p = 0.294		p = 0.167		p = 0.682		p = 0.979
Single^6^	121/140 (86%)	1	129/141 (91%)	1	106/14 0(76%)	1	31/123 (25%)	1
Ever married^6^	142/173 (82%)	0.72 (0.39–1.34)	148/174 (85%)	0.53 (0.23–1.09)	126/171 (74%)	0.90 (0.54–1.50)	37/159 (23%)	0.90 (0.52–1.56)
***BEHAVIOURAL FACTORS***								
**Age at first vaginal sex, years** ^7^		p = 0.601		p = 0.561		p = 0.914		p = 0.914
≤ 15	67/77 (87%)	1	66/78 (85%)	1	56/76 (74%)	1	15/70 (21%)	1
16–17	61/71 (86%)	0.91 (0.35–2.34)	62/71 (87%)	1.25 (0.49–3.18)	54/71 (76%)	1.13 (0.54–2.39)	16/63 (25%)	1.13 (0.54–2.39)
≥ 18	126/153 (82%)	0.70 (0.32–1.53)	137/153 (90%)	1.56 (0.70–3.48)	115/151 (76%)	1.14 (0.61–2.15)	37/137 (27%)	1.14 (0.61–2.15)
**Age difference between first vaginal sex partner and participant, years** ^8^		p = 0.134		**p = 0.046**		**p = 0.048**		p = 0.871
0	14/21 (67%)	1	15/21 (71%)	**1**	13/21 (62%)	**1**	5/18 (28%)	1
1–5	119/143 (84%)	2.59 (0.94–7.11)	123/143 (86%)	**2.46 (0.85–7.09)**	98/141 (70%)	**1.40 (0.54–3.63)**	32/128 (25%)	0.87 (0.29–2.62)
≥ 6	86/100 (86%)	3.07 (1.05–8.94)	92/100 (92%)	**4.60 (1.40–15.13)**	80/98 (82%)	**2.74 (0.99–7.57)**	25/89 (28%)	1.02(0.33–3.14)
**Number of vaginal sex partners in past three months** ^9^		p = 0.406		p = 0.861		p = 0.339		p = 0.492
1–25	27/36 (75%)	1	32/37 (86%)	1	25/36 (69%)	1	8/33 (24%)	1
26–50	62/72 (86%)	2.07 (0.75–5.66)	63/72 (88%)	1.09 (0.34–3.53)	50/71 (70%)	1.05 (0.44–2.51)	9/57 (16%)	0.59 (0.20–1.70)
51–75	26/29 (90%)	2.89 (0.70–11.87)	25/29 (86%)	0.98 (0.24–4.02)	22/29 (76%)	1.38 (0.46–4.19)	6/28 (21%)	0.85 (0.26–2.84)
76–100	57/66 (86%)	2.11 (0.75–5.92)	62/67 (93%)	1.94 (0.52–7.19)	48/67 (72%)	1.11 (0.46–2.70)	18/63 (29%)	1.25 (0.48–3.28)
>100	51/64 (80%)	1.31 (0.50–3.45)	53/64 (83%)	0.75 (0.24–2.36)	52/62 (84%)	2.29 (0.86–6.10)	16/59 (27%)	1.16 (0.44–3.10)
**Condom use at last vaginal sex** ^10^		p = 0.540		p = 0.927		p = 0.764		p = 0.509
No	16/18 (89%)	1	16/18 (89%)		14/18 (78%)		5/16 (31%)	1
Yes	246/294 (84%)	0.64 (0.14–2.88)	261/296 (88%)	0.93 (0.21–4.23)	218/292 (75%)	0.84 (0.27–2.64)	63/265 (24%)	0.69 (0.23–2.05)
**Ever gave oral sex to a male sexual partner**		p = 0.218		p = 0.127		p = 0.680		p = 0.780
No	215/252 (85%)	1	227/254 (89%)	1	186/251 (74%)	1	56/229 (24%)	1
Yes	48/61 (79%)	0.64 (0.31–1.29)	50/61 (82%)	0.54 (0.25–1.16)	46/60 (77%)	1.15 (0.59–2.23)	12/53 (23%)	0.90 (0.44–1.84)
**Ever received oral sex from a male sexual partner**		p = 0.287		p = 0.101		p = 0.504		p = 0.898
No	153/178 (86%)	1	163/180 (91%)	1	131/179 (73%)	1	40/164 (24%)	1
Yes	110/135 (81%)	0.72 (0.39–1.32)	114/135 (84%)	0.57 (0.29–1.12)	101/132 (77%)	1.19 (0.71–2.01)	28/118 (24%)	0.96 (0.55–1.68)
**Age initiated into sex work, years**		**p = 0.017**		**p<0.001**		p = 0.248		p = 0.176
≤19	21/24 (88%)	1	97/101 (96%)	**1**	20/24 (83%)	1	7/23 (30%)	1
20–24	69/77 (90%)	1.23 (0.30–5.07)	90/100 (90%)	**0.37 (0.11–1.23)**	58/77 (75%)	0.61 (0.19–2.01)	18/64 (28%)	0.89 (0.32–2.54)
25–29	85/100 (85%)	0.81 (0.21–3.06)	90/114 (79%)	**0.15 (0.05–0.46)**	78/99 (79%)	0.74 (0.23–2.41)	25/89 (28%)	0.89 (0.33–2.43)
≥30	88/112 (79%)	0.52 (0.14–1.91)			76/111 (68%)	0.43 (0.14–1.37)	18/106 (17%)	0.47 (0.17–1.30)
**Duration in sex work, years**		p = 0.539		p = 0.092		p = 0.494		p = 0.413
<1	63/73 (86%)	1	67/74 (91%)	1	59/74 (80%)	1	12/62 (19%)	1
1-<3	114/134 (85%)	0.90 (0.40–2.05)	122/134 (91%)	1.06 (0.40–2.83)	103/133 (77%)	0.87 (0.43–1.75)	33/124 (27%)	1.51 (0.72–3.18)
3-<5	50/59 (85%)	0.88 (0.33–2.34)	46/59 (78%)	0.37 (0.14–1.00)	36/57 (63%)	0.44 (0.20–0.95)	14/52 (27%)	1.54 (0.64–3.70)
≥5	36/47 (77%)	0.52 (0.20–1.34)	42/48 (88%)	0.73 (0.23–2.33)	34/47 (72%)	0.66 (0.28–1.56)	9/44 (20%)	1.07 (0.41–2.82)
**Ever had mutual masturbation** ^11^		p = 0.507		p = 0.308		p = 0.445		p = 0.404
No	6/8 (75%)	1	6/8 (75%)	1	5/8 (63%)	1	1/8 (13%)	1
Yes	257/305 (84%)	1.78 (0.35–9.11)	271/307 (88%)	2.51 (0.49–12.91)	227/303 (75%)	1.79 (0.42–7.68)	67/274 (24%)	2.27 (0.27–18.7)
**Female genital mutilation** ^12^		**p = 0.011**		p = 0.911		p = 0.168		p = 0.407
No	162/183 (89%)	**1**	163/185 (88%)	1	141/182 (77%)	1	42/162 (26%)	1
Yes	101/130 (78%)	**0.45 (0.24–0.83)**	114/130 (88%)	0.96 (0.48–1.91)	91/129 (71%)	0.70 (0.42–1.16)	26/120 (22%)	0.79 (0.45–1.38)
**Ever drank alcohol**		p = 0.401		p = 0.321		p = 0.343		p = 0.719
No	67/77 (87%)	1	71/78 (91%)	1	55/78 (71%)	1	18/70 (26%)	1
Yes	196/236 (83%)	0.73 (0.35–1.54)	206/237 (87%)	0.66 (0.28–1.55)	177/233 (76%)	1.32 (0.75–2.34)	50/212 (24%)	0.89 (0.48–1.66)
**Ever smoke cigarettes**		p = 0.776		p = 0.658		p = 0.375		p = 0.879
No	179/212 (84%)	1	187/214 (87%)	1	155/212 (73%)	1	47/197 (24%)	1
Yes	84/101 (83%)	0.91 (0.48–1.73)	90/101(89%)	1.18 (0.56–2.49)	77/99 (78%)	1.29 (0.73–2.26)	21/85 (25%)	1.05 (0.58–1.89)
**Ever taken any illicit drug** ^13^		p = 0.759		p = 0.632		p = 0.310		p = 0.978
No	200/237 (85%)	1	209/239 (87%)	1	172/235 (73%)	1	52/216 (24%)	1
Yes	63/76 (83%)	0.90 (0.45–1.79)	68/76 (89%)	1.22 (0.53–2.79)	60/76 (79%)	1.37 (0.74–2.56)	16/66 (24%)	1.01 (0.53–1.92)
**Ever had an STI**		p = 0.265		p = 0.827		p = 0.873		p = 0.594
**No**	227/267 (85%)	1	237/269 (88%)	1	198/266 (74%)	1	57/242(24%)	1
**Yes**	36/46 (78%)	0.63 (0.29–1.38)	40/46 (87%)	0.90 (0.35–2.29)	34/45(76%)	1.06(0.51–2.21)	11/40(28%)	1.23 (0.58–2.62)
**Ever heard of HPV**		p = 0.224		p = 0.088		p = 0.098		**p = 0.047**
No	253/299 (85%)	1	267/301(89%)	1	225/298 (76%)	1	62/270 (23%)	**1**
Yes	10/24 (71%)	0.45 (0.14–1.51)	10/14 (71%)	0.32 (0.09–1.07)	7/13 (54%)	0.38 (0.12–1.16)	6/12 (50%)	**3.35 (1.04–10.8)**
***BIOLOGICAL FACTORS***								
**RDT HIV final test result**		**p = 0.003**		p = 0.084		p = 0.061		**p = 0.011**
Negative	224/273 (82%)	**1**	237/273 (87%)	1	196/269 (73%)	1	52/243 (21%)	**1**
Positive	39/40 (96%)	**8.53 (1.14–63.60)**	40/42 (95%)	3.04 (0.70–13.11)	36/42 (86%)	2.23 (0.90–5.52)	16/39 (41%)	**2.56 (1.26–5.19)**
**Cervical HPV infection** ^14^	NA	NA		**p<0.001**		**p<0.001**		**p = 0.023**
No			29/50 (58%)	**1**	23/50 (46%)	**1**	5/44(11%)	**1**
Yes			246/263 (94%)	**10.48 (4.97–22.10)**	207/259 (80%)	**4.67 (2.48–8.81)**	62/236(26%)	**2.78 (1.05–7.37)**
**Vulvar HPV detection**		**p<0.001**	NA	NA		**p<0.001**		p = 0.083
No	17/38 (45%)	**1**			9/36 (25%)	**1**	4/32 (13%)	1
Yes	246/275 (89%)	**10.48 (4.97–22.10)**			223/275 (81%)	**12.87 (5.71–29.0)**	64/250 (26%)	2.41(0.81–7.13)
**Anal HPV detection** ^15^		**p<0.001**		**p<0.001**	NA	NA		**p = 0.043**
No	52/79 (66%)	**1**	52/79 (66%)	**1**			10/66 (15%)	**1**
Yes	207/230 (90%)	**4.67 (2.48–8.81)**	223/232 (96%)	**12.87 (5.71–28.99)**			57/212 (27%)	**2.06 (0.98–4.31)**
**Oral HPV detection** ^16^		**p = 0.023**		p = 0.083		**p = 0.043**	NA	NA
No	174/213 (82%)	**1**	186/214 (87%)	1	155/211 (73%)	**1**		
Yes	62/67 (93%)	**2.78 (1.05–7.37)**	64/68 (94%)	2.41 (0.81–7.13)	57/67 (85%)	**2.06 (0.98–4.31)**		

^**1**^-p-values were obtained from Wald tests;

^**2**^**-**others—Igbo, Hausa/Fulani and other ethnic minorities;

^**3**^**-**no religion—one participant said she had no religion;

^**4**^**-**others-seamstress(tailor), petty trading and teaching;

^**5**^–N—Naira-Nigeria currency; USD—United States Dollar;

^**6**^– Living alone;

^**7**^**-**N = 301–12 participants did not provide information on age at first vaginal sex;

^**8**^- N = 263–50 participants did not provide information to calculate age difference between first vaginal sex partner and participant;

^**9**^-N = 267–46 participants did not provide information on number of vaginal sex partners in past three months;

^**10**^-N = 312-one participant did not provide information on condom use during her last vaginal sex;

^**11**^-Mutual masturbation question was ‘have you or your partner ever touched each other’s genital area by hand? (Yes or No);

^**12**^-Female genital mutilation was based on the clinical examination of the female external genitalia for evidence of genital circumcision by the research nurse at the clinic (Yes or No);

^**13**^**-**Illicit drugs are banned substances or drugs taken by participants for non-medical reasons in Nigeria;

^**14**^-N = 313-three participants did not have cervical HPV results;

^**15**^-N = 309-four participants did not have anal HPV results;

^**16**^-N = 280–33 participants did not have oral HPV results;

**NA**- not applicable.

**Table 4:** The adjusted analysis of factors associated with cervical, vulvar, anal and oral human papillomavirus infection among brothel-based female sex workers in Ibadan, Nigeria.

Variable	Cervical	Vulvar	Anal	Oral
	p-value^1^ Adjusted OR (95%CI)^17^	p-value^1^ Adjusted OR (95%CI)^17^	p-value^1^ Adjusted OR (95%CI)^17^	p-value^1^ Adjusted OR (95%CI)^17^
***SOCIO-DEMOGRAPHIC FACTORS***				
**Age group, years**	**p = 0.035**	**p<0.001**	**p = 0.052**	p = 0.120
18–24	**1**	**1**	**1**	1
25–34	**0.44 (0.15–1.31)**	**0.16 (0.02–1.22)**	**0.39 (0.16–0.93)**	0.59 (0.29–1.21)
35–45	**0.26 (0.08–0.82)**	**0.07 (0.01–0.52)**	**0.37 (0.14–0.93)**	0.42 (0.18–0.96)
**Ethnicity**	p = 0.528	p = 0.164	p = 0.080	p = 0.831
Yoruba	1	1	1	1
Other^2^	0.71 (0.23–2.15)	0.39 (0.09–1.73)	0.43 (0.15–1.18)	1.11 (0.43–2.88)
**Religion**	p = 0.879	p = 0.709	p = 0.557	p = 0.928
Christianity	1	1	1	1
Islam and no religion^3^	0.93 (0.33–2.58)	1.27 (0.36–4.51)	1.32 (0.51–3.45)	1.04 (0.42–2.59)
**Highest education level**	p = 0.856	p = 0.314	p = 0.514	p = 0.534
No formal education	1	1	1	1
Primary	1.70 (0.45–6.36)	3.15 (0.75–13.28)	2.09 (0.68–6.43)	0.42 (0.14–1.27)
Secondary	1.36 (0.42–4.39)	2.27 (0.66–7.74)	1.40 (0.52–3.82)	0.42 (0.16–1.13)
Tertiary	1.14 (0.27–5.00)	1.18 (0.26–5.36)	1.92 (0.48–7.67)	0.65 (0.19–2.26)
**Quranic education**	p = 0.300	p = 0.662	p = 0.445	p = 0.640
No	1	1	1	1
Yes	0.55 (0.19–1.62)	1.39 (0.30–6.36)	1.54 (0.49–4.82)	0.77 (0.25–2.39)
**Other occupation**	p = 0.473	p = 0.603	p = 0.419	p = 0.852
Student or Apprentice	1	1	1	1
Others^4^	0.78 (0.40–1.52)	0.82 (0.39–1.74)	0.78 (0.44–1.41)	0.94 (0.50–1.78)
**Income per month** ^5^	p = 0.657	p = 0.819	**p = 0.030**	p = 0.515
≤20,000N (≤56 USD)	1	1	**1**	1
20,001–40,000N (>56–112USD)	0.94 (0.43–2.02)	1.24 (0.53–2.90)	**2.07 (1.11–3.86)**	0.69 (0.35–1.36)
> 40,000N (>112USD)	0.71 (0.33–1.55)	0.96 (0.41–2.28)	**2.10 (1.09–4.04)**	0.73 (0.37–1.44)
**Current marital status**	p = 0.300	p = 0.691	p = 0.424	p = 0.619
Single^6^	1	1	1	1
Ever married^6^	0.41 (0.11–1.41)	0.86 (0.40–1.84)	1.26 (0.72–2.22)	1.17 (0.63–2.15)
***BEHAVIOURAL FACTORS***	**Adjusted OR (95%CI)** ^18^	**Adjusted OR (95%CI)** ^18^	**Adjusted OR (95%CI)** ^18^	**Adjusted OR (95%CI)** ^18^
**Age at first vaginal sex, years** ^7^	p = 0.702	p = 0.338	p = 0.864	p = 0.670
≤ 15	1	1	1	1
16–17	0.89 (0.34–2.32)	1.52 (0.57–4.06)	1.15 (0.53–2.48)	1.22 (0.54–2.76)
≥ 18	0.73 (0.33–1.61)	1.89 (0.82–4.36)	1.20 (0.62–2.31)	1.37 (0.68–2.77)
**Age difference between first vaginal sex partner and participant, years** ^8^	p = 0.345	p = 0.121	p = 0.134	p = 0.806
0	1	1	1	1
1–5	1.98 (0.68–5.71)	1.52 (0.50–4.65)	0.98 (0.36–2.70)	0.68 (0.21–2.17)
≥ 6	2.32 (0.77–7.05)	3.18 (0.92–11.02)	1.83 (0.63–5.33)	0.74 (0.23–3.)
**Number of vaginal sex partners in past three months** ^9^	p = 0.489	p = 0.566	p = 0.546	p = 0.440
1–25	1	1	1	1
26–50	1.60 (0.56–4.55)	0.79 (0.23–2.70)	0.74 (0.29–1.90)	0.53 (0.17–1.63)
51–75	2.46 (0.58–10.43)	0.71 (0.16–3.13)	1.06 (0.32–3.48)	0.89 (0.26–3.10)
76–100	1.97 (0.69–5.67)	1.57 (0.41–5.99)	0.88 (0.35–2.21)	1.28 (0.47–3.50)
>100	1.05 (0.39–2.87)	0.60 (0.18–2.02)	1.59 (0.55–4.61)	0.96 (0.35–2.69)
**Condom use at last vaginal sex** ^10^	p = 0.432	p = 0.770	p = 0.844	p = 0.627
No	1	1	1	1
Yes	0.56 (0.12–2.61)	0.79 (0.16–3.87)	0.89 (0.27–2.91)	0.79 (0.24–2.36)
**Ever gave oral sex to a male sexual partner**	p = 0.175	p = 0.078	p = 0.845	p = 0.475
No	1	1	1	1
Yes	0.60 (0.29–1.24)	0.47 (0.22–1.06)	1.07 (0.54–2.15)	0.77 (0.37–1.61)
**Ever received oral sex from a male sexual partner**	p = 0.254	p = 0.307	p = 0.468	p = 0.790
No	1	1	1	1
Yes	0.70 (0.37–1.30)	0.68 (0.32–1.43)	1.22 (0.71–2.10)	1.09 (0.58–2.02)
**Age initiated into sex work, years**	p = 0.825		p = 0.175	p = 0.397
≤19	1	p = 0.171	1	1
20–24	2.12 (0.42–10.54)	1	1.07 (0.28–4.07)	1.16 (0.38–3.56)
25–29	2.13 (0.38–12.04)	0.65 (0.17–2.52)	2.39 (0.55–10.33)	1.47 (0.38–5.68)
≥30	1.77 (0.29–10.78)	0.29 (0.07–1.22)	1.19 (0.26–5.38)	0.77 (0.17–3.43)
**Duration in sex work, years**	p = 0.814	p = 0.356	p = 0.434	p = 0.396
<1	1	1	1	1
1-<3	1.00 (0.43–2.31)	1.16 (0.43–3.16)	0.91 (0.44–1.86)	1.59(0.72–3.48)
3-<5	1.18 (0.43–3.25)	0.58 (0.21–1.64)	0.54 (0.24–1.23)	0.99(0.32–3.06)
≥5	0.73 (0.27–1.97)	1.35 (3.99–4.60)	0.73 (0.30–1.81)	
**Ever had mutual masturbation** ^11^	p = 0.636	p = 0.465	p = 0.483	p = 0.444
No	1	1	1	1
Yes	1.53 (0.28–8.47)	2.00 (0.34–11.94)	1.76 (0.37–8.32)	2.16 (0.25–18.38)
**Female genital mutilation** ^12^	**p = 0.019**	p = 0.777	p = 0.327	p = 0.452
No	**1**	1	1	1
Yes	**0.48 (0.26–0.89)**	1.11 (0.54–2.26)	0.77 (0.45–1.30)	0.80 (0.45–1.42)
**Ever drank alcohol**	p = 0.642	p = 0.728	p = 0.446	p = 0.722
No	1	1	1	1
Yes	0.84 (0.39–1.80)	0.86 (0.35–2.09)	1.27 (0.69–2.35)	0.89 (0.47–1.69)
**Ever smoke cigarettes**	p = 0.660	p = 0.460	p = 0.412	p = 0.637
No	1	1	1	1
Yes	0.86 (0.45–1.67)	1.33 (0.62–2.88)	1.28 (0.71–2.33)	1.16 (0.63–2.13)
**Ever taken any illicit drug** ^13^	p = 0.406	p = 0.923	p = 0.697	p = 0.813
No	1	1	1	1
Yes	0.73 (0.36–1.51)	1.04 (0.44–2.47)	1.14 (0.59–2.17)	0.92 (0.48–1.79)
**Ever had an STI**	p = 0.296	p = 0.661	p = 0.883	p = 0.596
**No**	1	1	1	1
**Yes**	0.65 (0.29–1.44)	0.80 (0.30–2.11)	1.06 (0.50–2.26)	1.23 (0.57–2.66)
**Ever heard of HPV**	p = 0.283	p = 0.165	p = 0.147	**p = 0.029**
No	1	1	1	**1**
Yes	0.49 (0.14–1.69)	0.39 (0.11–1.36)	0.42 (0.13–1.33)	**3.88 (1.19–12.64)**
***BIOLOGICAL FACTORS***	**Adjusted OR (95%CI)** ^19^	**Adjusted OR (95%CI)** ^19^	**Adjusted OR (95%CI)** ^19^	**Adjusted OR (95%CI)** ^19^
**RDT HIV final test result**	**p = 0.014**	p = 0.545	p = 0.644	**p = 0.027**
Negative	**1**	1	1	**1**
Positive	**11.39 (1.03–125.83)**	1.66 (0.30–9.09)	1.27 (0.80–4.84)	**2.40 (1.12–5.14)**
Cervical HPV infection^14^	NA	**p<0.001**	p = 0.151	p = 0.307
No		**1**	1	1
Yes		**6.48 (2.70–15.57)**	1.97 (0.80–4.84)	1.75 (0.57–5.29)
**Vulvar HPV detection**	**p<0.001**	NA	**p<0.001**	p = 0.503
No	**1**		**1**	1
Yes	**16.60 (5.80–47.54)**		**10.55 (3.67–30.31)**	1.63 (0.37–7.11)
**Anal HPV detection** ^15^	p = 0.281	**p<0.001**	NA	p = 0.349
No	1	**1**		1
Yes	1.65 (0.69–3.99)	**8.88 (3.66–23.28)**		1.47(0.65–3.34)
**Oral HPV detection** ^16^	p = 0.262	p = 0.161	p = 0.206	NA
No	1	1	1	
Yes	1.89 (0.59–6.01)	2.08 (0.69–6.27)	1.69 (0.73–3.89)	

^**1**^-p-values were obtained from Wald tests;

^**2**^**-**others—Igbo, Hausa/Fulani and other ethnic minorities;

^**3**^**-**no religion—one participant said she had no religion;

^**4**^**-**others-seamstress(tailor), petty trading and teaching;

^**5**^–N—Naira-Nigeria currency; USD—United States Dollar;

^**6**^– Living alone;

^**7**^**-**N = 301–12 participants did not provide information on age at first vaginal sex;

^**8**^- N = 263–50 participants did not provide information to calculate age difference between first vaginal sex partner and participant;

^**9**^-N = 267–46 participants did not provide information on number of vaginal sex partners in past three months;

^**10**^-N = 312-one participant did not provide information on condom use during her last vaginal sex;

^**11**^-Mutual masturbation question was ‘have you or your partner ever touched each other’s genital area by hand? (Yes or No);

^**12**^-Female genital mutilation was based on the clinical examination of the female external genitalia for evidence of genital circumcision by the research nurse at the clinic (Yes or No);

^**13**^**-**Illicit drugs are banned substances or drugs taken by participants for non-medical reasons in Nigeria;

^**14**^-N = 313-three participants did not have cervical HPV results;

^**15**^-N = 309-four participants did not have anal HPV results;

^**16**^-N = 280–33 participants did not have oral HPV results;

^**17**^- Level 1 factors were adjusted for age and study site and other level 1 factors that were significant at p-value<0.10;

^**18**^- Level 2 factors were adjusted for age and study site (core variables from Level 1), other factors significant at level 1, and other level 2 factors that were significant at p-value<0.10;

^**19**^- Level 3 factors were adjusted for (core variables from Level 1), other level 1 factors, level 2 factors that were significant at p-value<0.10, and various biological factors, such as the detection of HPV genotype in the cervical, vulvar, anal and oral cavities of the participant;

**NA**- not applicable.

Only age of participants and presence of any cervical and anal HPV were independently associated with detection of vulvar HPV. The odds of detecting any vulvar HPV was lower among women aged 25–34 years (AOR = 0.16 (95% CI, 0.02–1.22) and 35–45 years (AOR = 0.07; 95% CI, 0.01–0.52) compared to those aged 18–24 years. Detection of vulvar HPV was associated with higher odds of having any anal (aOR = 8.88, 95% CI, 3.66–23.28) and cervical (aOR = 6.48, 95% CI, 2.70–15.57) HPV.

Factors associated with detection of any anal HPV include age, income and detection of any vulvar HPV. FSWs aged 25–34 years and 36–45 years had 0.39 (95% CI, 0.16–0.93) and 0.37 (95% CI, 0.14–0.93) odds of having any anal HPV, respectively, relative to those aged 18–24 years. The odds of having anal HPV was higher among those that earned 20,001 to 40,000 Naira (aOR = 2.07; 95% CI, 1.11–3.86) and more than 40,000 Naira (aOR = 2.10; 95% CI, 1.09–4.04) relative to those that earned less than 20,000 Naira a month. Reported anal sex was not considered as an explanatory factor for anal HPV due to small number of observations. FSWs that had any vulvar HPV had higher odds (aOR = 10.55, 95% CI, 3.67–30.31) of having any anal HPV than those without vulvar HPV. There was higher odds of oral HPV among FSWs that had ever heard of HPV (aOR = 3.88, 95% CI, 1.19–12.64) compared to those with no information about HPV. HIV positive FSWs had 2.40 (95% CI, 1.12–5.14) times higher odds of having any oral HPV compared to those that were HIV negative.

### Concordance of genotype specific HPV infection

Results of concordance of specific HPV genotypes were presented in S1 and S2 Tables and Table 5. HPV-35 (9/86) was the highest HR-HPV concordant in the four anatomic sites followed by HPV-51 (4/65) and HPV-16, -52, -58 (3/72; 3/70; 3/74). HPV-42 was the commonest concordant LR-HPV in seven participants, followed by HPV-44 in five, HPV-70 in four and HPV-6/11/40/43 in three participants each. The concordance of HR and LR HPV in three anatomic sites was highest between cervical, vulval and anal sites relative to other three possible combined sites. HPV-68 (26/83) was the most concordant HR-HPV type while HPV-52 (25/70) and HPV-31 (23/50) and HPV-35 (23/86) ranked second and third respectively, between cervical, vulvar and anal sites. Only HPV-16 (18/72) and HPV-52 (1/70) were detected in the cervix, vulva and oral cavity.

**Table 5:** Proportion of HPV genotype specific concordance samples across the four anatomic sites of cervix, vulvar, anal and oral cavities among brothel-based female sex workers in Ibadan, Nigeria.

Specific HPV Genotype	HPV detection (Yes/No)	FSW with the same HPV genotype in all the 4 sites (%)	FSW with the same HPV genotype in any 3 sites (%)	FSW with the same HPV genotype in any 2 sites (%)	FSW with HPV genotype in 1 site only (%)
FSW with any HPV16	Yes (n = 72)	3/72 (4%)	18/72 (25%)	15/72 (21%)	36/72 (50%)
	No (n = 60)	-	-	-	-
FSW with any HPV 18	Yes (n = 39)	2/39 (5%)	11/39 (28%)	10/39 (26%)	16/39 (41%)
	No (n = 38)	-	-	-	-
FSW with any HPV 31	Yes (n = 50)	1/50 (2%)	23/50 (46%)	6/50 (12%)	20/50 (40%)
	No (n = 55)	-	-	-	-
FSW with any HPV 33	Yes (n = 20)	2/20 (10%)	5/20 (25%)	8/20 (40%)	5/20 (25%)
	No (n = 24)	-	-	-	-
FSW with any HPV 35	Yes (n = 86)	9/86 (11%)	23/86 (27%)	19/86 (22)	35/86 (41%)
	No (n = 92)	-	-	-	-
FSW with any HPV 39	Yes (n = 40)	2/40 (5%)	17/40 (43%)	8/40 (20%)	13/40 (33%)
	No (n = 48)	-	-	-	-
FSW with any HPV 45	Yes (n = 54)	0	21/54 (39%)	10/54 (19%)	23/54 (43%)
	No (n = 52)	-	-	-	-
FSW with any HPV 51	Yes (n = 65)	4/65 (6%)	17/65 (26%)	14/65 (22%)	30/65 (46%)
	No (n = 60)	-	-	-	-
FSW with any HPV 52	Yes (n = 70)	3/70 (4%)	25/70 (36%)	16/70 (23%)	26/70 (37%)
	No (n = 75)	-	-	-	-
FSW with any HPV 56	Yes (n = 47)	1/47 (2%)	13/47 (28%)	11/47 (23%)	22/47 (47%)
	No (n = 40)	-	-	-	-
FSW with any HPV 58	Yes (n = 74)	3/74 (4%)	19/74 (26%)	16/74 (22%)	36/74 (49%)
	No (n = 63)	-	-	-	-
FSW with any HPV 59	Yes (n = 48)	2/48 (4%)	17/48 (35%)	8/48 (17%)	21/48 (44%)
	No (n = 48)	-	-	-	-
FSW with any HPV 68	Yes (n = 83)	1/83 (1%)	26/83 (31%)	22/83 (27%)	34/83 (41%)
	No (n = 77)	-	-	-	-
FSW with any HPV 6	Yes (n = 54)	3/54 (6%)	8/54 (15%)	18/54 (33%)	25/54 (46%)
	No (n = 43)	-	-	-	-
FSW with any HPV 11	Yes (n = 25)	3/25 (12%)	4/25 (16%)	10/25 (40%)	8/25 (32%)
	No (n = 27)	-	-	-	-
FSW with any HPV 26	Yes (n = 13)	0	2/13 (15%)	7/13 (54%)	4/13 (31%)
	No (n = 11)	-	-	-	-
FSW with any HPV 40	Yes (n = 50)	3/50 (6%)	17/50 (34%)	12/50 (24%)	18/50 (36%)
	No (n = 55)	-	-	-	-
FSW with any HPV 42	Yes (n = 78)	7/78 (9%)	25/78 (32%)	22/78 (28%)	24/78 (31%)
	No (n = 93)	-	-	-	-
FSW with any HPV 43	Yes (n = 42)	3/42 (7%)	12/42 (29%)	11/42 (26%)	16/42 (38%)
	No (n = 44)	-	-	-	-
FSW with any HPV 44	Yes (n = 81)	5/81 (6%)	23/81 (28%)	24/81 (30%)	29/81 (36%)
	No (n = 85)	-	-	-	-
FSW with any HPV 53	Yes (n = 89)	2/89 (2%)	38/89 (43%)	26/89 (29%)	23/89 (26%)
	No (n = 108)	-	-	-	-
FSW with any HPV 54	Yes (n = 87)	2/87 (2%)	26/87 (30%)	25/87 (29%)	34/87 (39%)
	No (n = 83)	-	-	-	-
FSW with any HPV 61	Yes (n = 17)	0	1/17 (6%)	4/17 (24%)	12/17 (71%)
	No (n = 6)	-	-	-	-
FSW with any HPV 66	Yes (n = 37)	1/37 (3%)	11/37 (30%)	12/37 (32%)	13/37 (35%)
	No (n = 37)	-	-	-	-
FSW with any HPV 69	Yes (n = 33)	2/33 (6%)	7/33 (21%)	2/33 (6%)	22/33 (67%)
	No (n = 22)	-	-	-	-
FSW with any HPV 70	Yes (n = 57)	4/57 (7%)	20/57 (35%)	11/57 (19%)	22/57 (39%)
	No (n = 63)				
FSW with any HPV 73	Yes (n = 49)	2/49 (4%)	12/49 (25%)	16/49 (33%)	19/49 (39%)
	No (n = 46)	-	-	-	-
FSW with any HPV 82	Yes (n = 43)	1/43 (2%)	12/43 (28%)	12/43 (28%)	18/43 (42%)
	No (n = 39)	-	-	-	-

Light yellow—high-risk HPV genotypes and Blue—low-risk HPV genotype.

In the six possible two-sites comparisons, concordance of HR and LR-HPV was highest between cervix and vulva, and least between oral and anal cavity. HPV-68 (17/83) and HPV-35 (13/86) were the mostly frequently detected HR genotypes between cervix and vulvar sites (S2 Table). Of the 15 participants that had HPV-16 in any two sites, most were between cervix and vulvar. HPV-54 (20/87) and HPV-53 (19/89) were the two most frequently detected LR types between cervix and vulva.

## Discussion

In this study, HPV infection was highly prevalent with 96% of FSWs having at least 1 genotype-specific infection in one of the 4 sites. More than 70% of FSW had HPV in the vulva, cervix, and anus, and 24% had an oral HPV infection. Multiple HPV infections were also common, especially in the vulva. HPV-35 was the most common HR-HPV infections in the 4 anatomic sites. To our knowledge this is the largest data on the prevalence of, risk factor and concordance of HPV infections in the oral, genital and anal sites among FSWs.

The prevalence of any HPV, HR-and LR-HPV, and multiple HPV infections in the oral, cervical, vulvar and anal sites in this study was higher than the similar prevalences by anatomical sites reported among women in the general population in Ibadan, Nigeria [12]. This is similar to data from Hungary which showed that the prevalence of HPV infection was higher among FSWs compared to women in the general population for both cervical (64.0% versus 34.6%, p = 0.006) and anal (50.0% versus 15.4%, p = 0.001) samples with the exception of oral samples where the difference was not statistically significant (20.6% versus 7.7%, p = 0.103) [15].

Most studies reported on cervical HPV and fewer studies were on anal, vulvar, anal and oral HPV infections in FSWs [16–23]. The prevalence of cervical HPV in FSWs from countries in SSA, Europe and Asia was between 25–85% [18, 20, 21, 24–26]. Among studies with data on multiple anatomic sites, a study amongst 188 FSWs in Spain found that the overall HPV prevalence was lower than in our study and was most prevalent in the cervix (27.8%), followed by the vagina (26.1%), vulva (22.9%), anal cavity (15.0%) and oral cavity (7.9%) [27]. In the Togo study of 310 FSW, the prevalence of any HPV and HR-HPV was 45.2% and 32.9% in the cervix, and 34.8% and 20.7% in the anal cavity, respectively, which is similar to this study [10]. However, a higher (80.0%) prevalence of anal HPV infection, but a lower (50.0%) HR-HPV infection compared to this study was reported in the Netherlands study [28]. The high prevalence observed in the Netherlands study might be due to high proportion of FSWs with STI complaints in a routine STD clinic relative to the Togo study participants that were recruited at point of sex work service.

To date, this study presents the first data on oral HPV among FSWs in SSA. Oral HPV infection was higher in our study than two previous studies that reported oral HPV in FSW; 7.6% among 185 FSWs aged 18–26 years in Peru and 6.1% among 196 FSWs aged18-45 years in Japan [19, 23]. The observed difference in oral HPV prevalence might be due to differences of diagnostic tests that were used in previous studies and reported use of condom/barrier methods during oral sex. For example, 90% used condom during oral sex in the Peruvian study [23].

Contrary to previous studies that showed that HPV-16, HPV-31, HPV-51 or HPV-58 as the most frequent genotypes in the cervix and anus of FSWs in Europe and SSA [15, 20, 21], we found HPV-35 as the most common HR-HPV and concordant in the four anatomic sites. This finding is similar to our study among Nigerian women in the general population [12]. Unfortunately, HPV-35 is not covered in the three HPV vaccines despite the reported strong association with invasive cervical cancer in SSA relative to other regions [29].

The low level of awareness of HPV vaccination among our participants despite having high prevalence of HPV infections in multiple anatomic sites is worrisome. Previous Nigerian studies have also reported low level of awareness and knowledge of HPV vaccination in sexually active women [30, 31]. Though, HPV vaccine has been approved for individual use, but it is yet to be launched and incorporated into the National immunization programme by the Government of Nigeria.

Similar to other studies, presence of HPV infection in a genital or anal sites as a common risk factor for detection of HPV infection in another anatomical site [10, 28]. Concordant HPV infection might be due to viral shedding between contiguous anatomic structures and or autoinoculation from sexual risk behaviours or unhygienic practices [32]. The concordant HPV infection of two or more anatomic sites may suggest the need for a screening protocol in multiple anatomic sites [32]. For example, an individual with a positive HPV DNA with premalignant lesion of the cervix may also be offered opportunity to screen for premalignant lesions of the anus.

HIV infection is another risk factor found to be associated with cervical and oral HPV infections in our study, as in previous studies [10, 18]. The association between HPV and HIV may be bi-directional and synergistic and HIV could be a risk factor for HPV acquisition and vice versa [33, 34]. Furthermore, HIV and HPV share similar risk factors for acquisition such as early age of sexual debut, multiple sexual partners, unprotected sex and the presence of other STIs [34]. Having a higher monthly income was associated with anal HPV infection, and this observed association might suggest that FSWs were engaging in high risk sexual behaviours in exchange for more money from their customers [35]. A Nigerian qualitative study reported that FSWs engaged in heterosexual anal and oral sex in exchange for a high fee from their clients, but this might have been underreported in this study due to stigma [36]. We could not explore anal sex due to low number of observations in this study. Other risk factors associated with cervical and anal HPV infections in previous studies were the presence of other STIs, multiple sexual partners, age of sexual partners, age of first vaginal sex, inconsistent condom or barrier methods, unprotected sex, intravaginal practices and illicit drug use [15, 25]. Oral HPV infection was also associated with oral sex and smoking in other studies [15, 23].

We observed a reduced risk of cervical HPV infection among FSWs with clinical evidence of FGM. It is plausible that the observed reduced risk might be due to less sexual activity from lack of sexual pleasure or orgasm and or increased sexual dysfunctions among people with FGM [37, 38]. However, a retrospective study of 2,398 women (18–90 years) in Senegal reported high risk of cervical cancer in women with FGM after adjusting for HIV and HPV infections and other benign and premalignant cervical lesions [39]. It was suggested that high levels of chronic inflammation around the areas affected by the mutilation might be a precursor for HPV acquisition and persistence [40]. In a case control study in Mali, women that reported type 1 FGM (partial circumcision) had higher odds (OR = 14.8, 95% CI: 4.14–896) of having cervical cancer relative to the controls [41].

The major strength of this study was the collection of multiple HPV data at the same time, and this is the first study to date with such diverse anatomic sampling among FSWs. We used probability sampling to select participants, and also limited recruitment to FSWs in the brothels. Previous studies that combined different sub-groups of FSWs from brothels, entertainment venues and streets, while others provided no information on the sub-group of FSWs that were recruited [10, 17, 27]. The limitations include the cross-sectional design that does not allow us to determine causality of risk factors for HPV infection and to examine the time of HPV acquisition, clearance and persistence. The study excluded younger adolescent girls engaging in sex work. In addition, the diagnosis of an STI was based on clinical information only and this would have excluded those with asymptomatic STIs.

In conclusion, we found a high prevalence of HPV in FSW in Nigeria, and this is higher compared to similar studies among FSW in different settings. Similar to our previous study, HPV-35 was the most common HR-HPV in all sites. The study also showed evidence of high infection among HIV positive participants especially in the cervical and oral sites. Future studies should include other categories of FSWs such as those working on the streets and entertainment venues who might be even more vulnerable to HPV infection. Longitudinal studies will help to answer other critical issues such as the incidence, clearance, and persistence of HPV in this group. Over 60% of FSW had evidence of HR-HPV in the cervical, vulvar and anal sites and in over 60% of women aged 35 years and around 15% had HR-HPV in the oral cavity, highlighting the need for prophylactic HPV vaccination and screening in Nigeria.

## Supporting information

S1 Fig Conceptual framework of the risk factor analysis for any HPV infection among female sex workers in Ibadan Nigeria. (DOCX)

S2 Fig Prevalence of specific cervical, vulvar, anal, and oral HPV genotypes among female sex workers in Ibadan, Nigeria. (DOCX)

S1 Table Pattern of HPV concordance by means of anatomical sites among brothel-based female sex workers in Ibadan, Nigeria. (DOCX)

S2 Table Concordance of genotype specific HPV infections across the four anatomic sites. (XLSX)
