# Supplementary material for: Oral, genital and anal human papillomavirus infections among female sex workers in Ibadan, Nigeria
Source: PLoS One. 2022 Mar 30;17(3):e0265269. doi: 10.1371/journal.pone.0265269 (PMC8967011; doi:10.1371/journal.pone.0265269)
Supplement: S1 Fig — (DOCX) [file pone.0265269.s001.docx]

**S1Figure: Conceptual Framework of the risk factor analysis for any HPV infection among female sex workers in Ibadan Nigeria**

**OUTCOME MEASURE**

**BIOLOGICAL DETERMINANT**

(LEVEL 3 FACTORS)

**PROXIMATE DETERMINANT**

(LEVEL 2 FACTORS)

**UNDERLYING DETERMINANT**

(LEVEL 1 FACTORS)

SEXUAL BEHAVIOUR CHARACTERISTICS

Age at 1^st^ vaginal sex (year)

Age difference between 1^st^ vagina sex partner and participant (year)

No of vaginal sex partners/three months

Ever gave oral sex (Yes/No); Ever received oral sex (Yes/No)

Ever cleansed inside vaginal (Yes/No)

Mutual masturbation (Yes/No)

**SOCIO-DEMOGRAPHICS**

Age group

Ethnicity

Religion

Highest education

Quranic education

Occupation aside sex work

Monthly income

Current marital status

**RAPID HIV final test result**

[Positive/Negative]

**HPV INFECTION AT OTHER ANATOMIC SITES APART FROM OUTCOME MEASURE** (Yes/No)

**HPV BY ANATOMIC SITES**

1) Any Cervical HPV

2) Any Vulva HPV

3) Any Anal HPV

4) Any Oral HPV

SEX WORK RELATED FACTORS

Age initiated to sex work (year)

Duration in the sex work business (year)

CULTURE/SOCIAL FACTORS

Female genital mutilation (Yes/No)

Ever drank alcohol (Yes/No)

Ever smoked cigarette (Yes/No)

Ever taken Illicit drugs

OTHER RELATED FACTORS

Ever had sexually transmitted infection

Ever heard of human papillomavirus
