# Supplementary material for: Oral, genital and anal human papillomavirus infections among female sex workers in Ibadan, Nigeria
Source: PLoS One. 2022 Mar 30;17(3):e0265269. doi: 10.1371/journal.pone.0265269 (PMC8967011; doi:10.1371/journal.pone.0265269)
Supplement: S2 Fig — (DOCX) [file pone.0265269.s002.docx]

**S2Figure: Prevalence of specific cervical, vulvar, anal and oral HPV genotypes among female sex workers in Ibadan, Nigeria**

**RED** triangle indicates HR-HPV point prevalence and **BLACK BALL** indicates LR-HPV point prevalence with lines indicating 95% CI
