# Supplementary material for: Oral, genital and anal human papillomavirus infections among female sex workers in Ibadan, Nigeria
Source: PLoS One. 2022 Mar 30;17(3):e0265269. doi: 10.1371/journal.pone.0265269 (PMC8967011; doi:10.1371/journal.pone.0265269)
Supplement: S1 Table — (DOCX) [file pone.0265269.s003.docx]

**S2Table 1: Pattern of HPV concordance by means of anatomical sites among brothel-based female sex workers in Ibadan, Nigeria (n=315)**

| **HPV Classification** | **Anatomic sites** | **Frequency (n/N)^1^** | **Percentage** |
| --- | --- | --- | --- |
| **Any HPV** |  |  |  |
|  | Cervical, anal, vulval and oral cavities | 54/315 | 17.1% |
|  | Cervical, anal and vulval cavity | 204/315 | 64.8% |
|  | Cervical, anal and oral cavity | 54/315 | 17.1% |
|  | Cervical, vulval and oral cavity | 61/315 | 19.4% |
|  | Anal, vulval and oral cavity | 57/315 | 18.1% |
|  | Cervical and anal cavity | 207/315 | 65.7% |
|  | Cervical and vulval cavity | 246/315 | 78.1% |
|  | Cervical and oral cavity | 62/315 | 19.7% |
|  | Anal and vulval cavity | 223/315 | 70.8% |
|  | Anal and oral cavity | 57/315 | 18.1% |
|  | Vulval and oral cavity | 64/315 | 20.3% |
| **Any HR-HPV** |  |  |  |
|  | Cervical, anal, vulval and oral cavities | 33/315 | 10.5% |
|  | Cervical, anal and vulval cavity | 154/315 | 48.9% |
|  | Cervical, anal and oral cavity | 33/315 | 10.5% |
|  | Cervical, vulval and oral cavity | 34/315 | 10.8% |
|  | Anal, vulval and oral cavity | 35/315 | 11.1% |
|  | Cervical and anal cavity | 157/315 | 49.8% |
|  | Cervical and vulval cavity | 196/315 | 62.2% |
|  | Cervical and oral cavity | 36/315 | 11.4% |
|  | Anal and vulval cavity | 173/315 | 54.9% |
|  | Anal and oral cavity | 36/315 | 11.4% |
|  | Vulval and oral cavity | 36/315 | 11.4% |
| **Any LR-HPV** |  |  |  |
|  | Cervical, anal, vulval and oral cavities | 30/315 | 9.5% |
|  | Cervical, anal and vulval cavity | 140/315 | 44.4% |
|  | Cervical, anal and oral cavity | 30/315 | 9.5% |
|  | Cervical, vulval and oral cavity | 35/315 | 11.1% |
|  | Anal, vulval and oral cavity | 31/315 | 9.8% |
|  | Cervical and anal cavity | 141/315 | 44.8% |
|  | Cervical and vulval cavity | 194/315 | 61.6% |
|  | Cervical and oral cavity | 35/315 | 11.1% |
|  | Anal and vulval cavity | 155/315 | 49.2% |
|  | Anal and oral cavity | 31/315 | 9.8% |
|  | Vulval and oral cavity | 36/315 | 11.4% |
